# Mitochondrial toxicity evaluation of traditional Chinese medicine injections with a dual *in vitro* approach

**DOI:** 10.3389/fphar.2022.1039235

**Published:** 2022-11-02

**Authors:** Yunfu Shen, Kaiqiang Guo, Aijun Ma, Zhe Huang, Jingjing Du, Junhe Chen, Qianyu Lin, Chengming Wei, Zi Wang, Fuming Zhang, Juan Zhang, Wanjun Lin, Na Feng, Wenzhe Ma

**Affiliations:** ^1^ State Key Laboratory of Quality Research in Chinese Medicine, Macau University of Science and Technology, Macao, Macao SAR, China; ^2^ School of Biotechnology and Health Sciences, Wuyi University, Jiangmen, China; ^3^ Antibody Engineering Laboratory, School of Life Science & Technology, China Pharmaceutical University, Nanjing, China

**Keywords:** TCM injections, mitochondrial toxicity, adverse drug reactions, glucose/galactose assay, HepG2 cells

## Abstract

There are technical obstacles in the safety evaluation of traditional Chinese medicine (TCM) injections due to their complex chemical nature and the lack of rapid and accurate *in vitro* methods. Here, we established a dual *in vitro* mitochondrial toxicity approach combing the conventional “glucose/galactose” assay in HepG2 cells with the cytotoxic assay in mitochondrial respiration deficient cells. Using this dual *in vitro* approach, for the first time, we systematically assessed the mitochondrial toxicity of TCM injections. Four of the 35 TCM injections, including Xiyanping, Dengzhanhuasu, Shuanghuanglian, and Yinzhihuang, significantly reduced cellular ATP production in galactose medium in the first assay, and presented less cytotoxic in the respiration deficient cells in the second assay, indicating that they have mitochondrial toxicity. Furthermore, we identified scutellarin, rutin, phillyrin, and baicalin could be the potential mitochondrial toxic ingredients in the 4 TCM injections by combining molecular docking analysis with experimental validation. Collectively, the dual *in vitro* approach is worth applying to the safety evaluation of more TCM products, and mitochondrial toxic TCM injections and ingredients found in this study deserve more attention.

## Graphical Abstract

**Figure:**
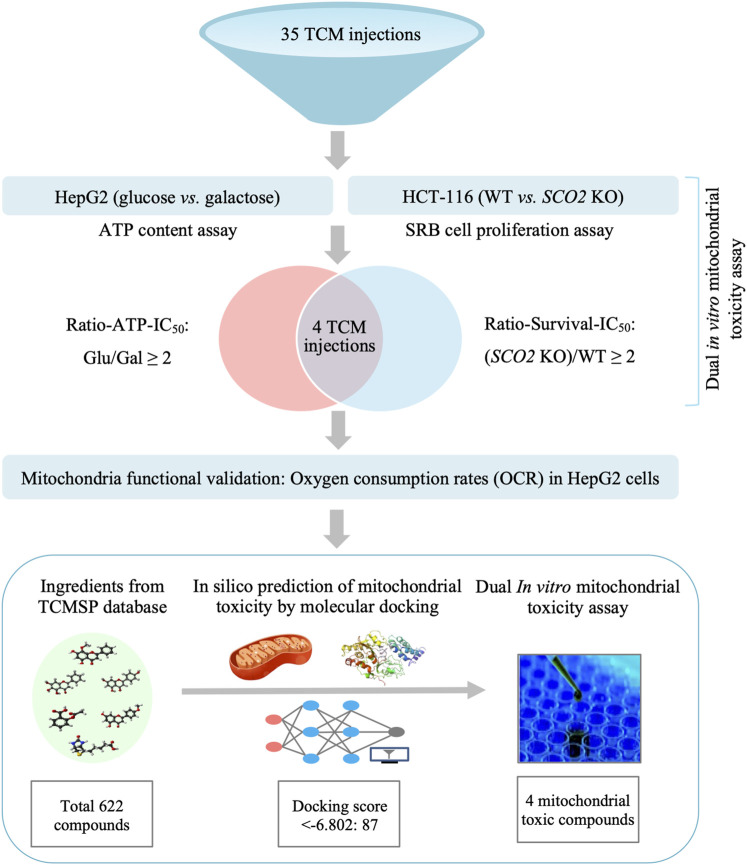
A dual in vitro mitochondrial toxicity assay approach combing the conventional “glucose/galactose” assay in HepG2 cells with the cytotoxic assay in mitochondrial respiration deficient cells was established in this study. Using this platform, we systematically assessed the mitochondrial toxicity of TCM injections for the first time. Four TCM injections were identified with potential mitochondrial toxicity. Their toxic ingredients were predicted by molecular docking and validated by the dual in vitro approach.

## Introduction

Traditional Chinese medicine (TCM) injections are a unique class of dosage forms consisting of active extracts from TCM products. TCM injections have been widely used in China since Chaihu (*Bupleurum*) injection was first developed in the 1940s, and have benefitted many patients, especially considering the cost-benefit tradeoffs (Xue 1984). However, increasing concerns have been raised regarding the safety of TCM injections in recent years. According to the Annual Report for National Adverse Drug Reaction Monitoring, adverse drug reactions (ADRs) caused by TCM injections accounted for 50% of all ADRs of TCMs in 2015-2015 (National Center for ADR Monitoring, 2015-2019). In contrast to conventional injections, TCM injections have a similar incidence of serious ADRs (6.02% *vs*. 6.72%) but a much higher incidence of unknown ADRs (46.74% *vs*. 24.13%) (Li et al., 2019). Therefore, significant efforts have been devoted to developing platforms for toxicity evaluation of TCMs using genomic, metabolomic, microtoxicity techniques, or animal models, which have been extensively reviewed elsewhere (Gao et al., 2019; Xie et al., 2019; Yu et al., 2020; He et al., 2021; Tu et al., 2021; Jiang et al., 2022; Wang et al., 2022). However, unlike chemical or biological drugs, the complexity of TCM ingredients makes safety assessment extremely difficult. Therefore, there is an unmet need for more explicit methods for toxicity testing of TCM injections.

Mitochondria are intracellular powerhouses that generate most of the ATP required for a cell’s biochemical reactions. They are also signaling hubs associated with thermogenesis, metabolite synthesis and transport, redox singling, calcium, copper and iron homeostasis, and cell death (Osellame et al., 2012; Mottis et al., 2019). Mitochondria are characterized by high lipid content in their membranes and proximity to respiratory byproducts of reactive oxygen species, leading them more vulnerable to chemical exposure (Meyer et al., 2018). Consequently, drugs that impede mitochondrial function can cause severe adverse effects. Approximately 35% of pharmaceutically relevant compounds are mitotoxic (Meyer and Chan 2017). Mitochondrial toxicity was the main cause of troglitazone-induced liver injury and cerivastatin-induced rhabdomyolysis, which ultimately led to the withdrawal of these two blockbusters from the market in 1997 and 2001, respectively (Hu et al., 2015; Huang et al., 2019). Drug-induced mitochondrial dysfunction has been observed in many different drug types, such as antivirals, antibiotics, chemotherapy agents, antipsychotics, antiepileptics, and antidepressants, which can lead to ADRs affecting the liver, muscle, kidney, and central nervous system (Stoker et al., 2019). However, mitochondrial toxicity is rarely detected in traditional preclinical animal models (Will and Dykens 2014). Therefore, a series of *in vitro* methods to detect mitochondrial toxicity have been developed over the past decade. One of the most widely employed high-throughput methods is called the “glucose/galactose” assay, in which glucose in the cell culture media is replaced with galactose, forcing the cells to produce ATP through oxidative phosphorylation (OXPHOS) rather than glycolysis and is therefore susceptible to mitochondrial toxicants (Swiss and Will 2011; Kamalian et al., 2015). In our studies, the human colorectal cancer cell line HCT116 *SCO2* KO is deficient in the mitochondrial complex IV assembly *SCO2* gene. Compared with the parental wild-type cells (HCT116 WT), HCT116 *SCO2* KO cells have insufficient respiratory function and are resistant to the inhibition of cell proliferation by the mitochondrial toxic drug metformin (Sung et al., 2010; Wang et al., 2017). Therefore, the correlation and the scientific basis for combining the two assays is that mitochondrial toxicants can decrease intracellular ATP levels in galactose medium in the first assay, while are less cytotoxic in HCT116 *SCO2* KO cells in the second assay.

Although herbal or TCM products are generally considered harmless, it has been aware in recent years that they also contain mitochondrial toxicants. *Ligularia dentat* Hara and *Ligularia hodgsonii* Hook herbs have been used for the treatment of cough, hepatitis, and inflammation in TCM. Their hepatotoxicity is caused by the high abundance of clivorine, an otonecine-type pyrrolizidine alkaloid that induces mitochondrial-dependent cell death (Ji et al., 2008; Ji et al., 2010). Neo-clerodane diterpenes present in the extensively used TCM herb skullcap are transformed into reactive metabolites by cytochrome P-450 in hepatocytes, which subsequently cause mitochondrial permeability transition, caspase activation, and apoptosis (Haouzi et al., 2000). The TCM herb Ephedra sinica, also known as Chinese ephedra or Ma Huang, induces mitophagy and inhibits mitochondrial biogenesis (Lee et al., 2017). The US Food and Drug Administration (FDA) banned the sale of ephedra-containing products, such as weight loss formulas and energy boosters, in 2004 due to cardiotoxicity (Zell-Kanter et al., 2015). However, compared with routine screening in the modern pharmaceutical industry, a systematic evaluation of the mitochondrial toxicity of TCM products is lacking.

In this study, we established a platform integrating two *in vitro* assays to enhance the prediction of mitochondrial toxicants. After the initial screen with the “glucose/galactose” assay, the resulting mitochondrial toxicants were counter-proved in a cell line deficient in mitochondrial respiration. Using this dual *in vitro* approach, for the first time, we systematically assessed mitochondrial toxicity of 35 TCM injections. Xiyanping (XYP), Dengzhanhuasu (DZHS), Shuanghuanglian (SHL), and Yinzhihuang (YZH) injections were identified as potential mitotoxicants which may correlate with their reported ADRs. Subsequently, the mitotoxic ingredients in the 4 TCM injections were identified by molecular docking and validated by functional assays.

## Materials and methods

### Drugs and reagents

TCM injections, TCM ingredients, rotenone, oligomycin A, antimycin A, metformin, as well as cytotoxic digoxin and tamoxifen were respectively purchased from commercial suppliers. Detailed information was shown Supplementary Tables S1, S2. Sulforhodamine B (SRB), Tris-base, puromycin, trichloroacetic acid (TCA), and crystal violet were obtained from Sigma Aldrich (St. Louis, MO, United States). Fetal bovine serum (FBS) and Dulbecco’s modified Eagle’s medium (DMEM) without glucose were obtained from Gibco (Brooklyn, NY, United States). Glucose and galactose were obtained from Psaitong (Beijing, China). HEPES was obtained from Aladdin (Shanghai, China). Sodium pyruvate was obtained from Innochem (Atlanta, GA, United States). CellTiter-Glo assay kit was purchased from Promega (Madison, WI, United States).

### Cell lines and cell culture

The HCT116 and HepG2 cell lines were purchased from the American Type Culture Collection (ATCC, Manassas, VA, United States), and the HCT116 *SCO2* KO cell line was a generous gift from Dr. Paul M. Hwang of the National Institutes of Health (NHLBI/NIH, United States). All three cell lines were cultured in DMEM supplemented with 1% antibiotics (0.1 mg/ml streptomycin and 100 U/ml penicillin) and 10% fetal bovine serum (FBS) at 37°C and 5% CO2 in a humidified incubator. The high-glucose media consisted of DMEM, containing 1 mM sodium pyruvate, 25 mM glucose, supplemented with 10% FBS, 1% antibiotics, and 5 mM N-2-hydro-xyethylpiperazine-N′-2-ethanesulfonic acid (HEPES). The galactose media is made up of DMEM supplemented with 10 mM galactose (without glucose), 5 mM HEPES, 1 mM sodium pyruvate, 10% FBS, and 1% streptomycin/penicillin as above.

### Glucose/galactose assay

The glucose/galactose assay was performed in HepG2 cells after treatment with TCM injections or ingredients as described previously (Swiss and Will 2011; Kamalian et al., 2015; Orlicka-Płocka et al., 2020). Briefly, HepG2 cells were seeded at a density of 1× 10^4^ cells/well in 96-well plates and cultured in either galactose or glucose medium. After 48 h of treatment with various concentrations of compounds or DMSO vehicle, total cellular adenosine triphosphate (ATP) content was examined with the CellTiter-Glo™ Luminescent Cell Viability Assay Kit from Promega (Madison, WI, United States) according to the manufacturer’s protocol. And the luminescence signal was measured by the SpectraMax paradigm microplate reader from Molecular Devices (Sunnyvale, CA, United States). The IC_50_ values, defined as the drug concentrations producing a 50% reduction in cellular ATP content, were calculated by fitting the data to the “log (inhibitor) *vs*. response --Variable slope (four parameters)” equation.

### Cell proliferation assay

The antiproliferative activity of TCM injections and ingredients on HCT116 WT and HCT116 *SCO2* KO cell lines were determined by the sulforhodamine B (SRB) colorimetric assay as described previously (Huang et al., 2021). In brief, cells were inoculated in 96-well plates in a volume of 90 μl/well at densities of 5,000 (HCT116 WT) or 15,000 (HCT116 *SCO2* KO) cells/well. Next, cells were incubated at 37°C and 5% CO_2_ in a humidified incubator overnight before adding a 10 μl medium containing compounds (10X indicated concentrations). After 72 h of treatment, cells were fixed by 50 μl cold 50% (w/v) TCA at 4°C for 1 h and then stained with 100 μl 0.4% (w/v) SRB. The protein-bound dye was solubilized with 200 μl 10 mM Tris-base solution (pH 10.5), and the absorbance at 515 nm was determined using the SpectraMax paradigm microplate reader. The IC_50_ value was defined as a concentration that inhibited cell growth by 50% and calculated using the equation of the “log (inhibitor) *vs*. response --Variable slope (four parameters)”

### Cellular mitochondrial respiratory function detection

The mitochondrial respiratory activity of HepG2 cells was determined using a fluorescence lifetime micro-oxygen monitoring system from Instech instruments (Plymouth, PA, United States) at 25°C according to the product manual. Briefly, HepG2 cells were inoculated in 6-well plates at a volume of 2500 μl/well at densities of 1 × 10^6^−5 × 10^6^ cells/well and incubated overnight. Then, cells were treated with TCM injections or ingredients at indicated concentrations for 24 h. Changes in %O_2_ per minute in HepG2 cells were measured according to the manufacturer’s protocol by Fluorescence Lifetime Micro Oxygen Monitoring System. The oxygen consumption rate (OCR) was calculated by the slope of the O_2_ data using linear regression and the results were reported as %O_2_ per minute, normalized to the number of cells.

### Molecular docking simulation

The 3D structure of the target protein NDUFV1 (PDB ID: 5LC5), a complex composed of multiple subunits, was collected from the Protein Data Bank (PDB) (https://www.rcsb.org/). The structure files of compounds were derived from the traditional Chinese medicine systems pharmacology (TCMSP) database (https://tcmsp-e.com/) and uploaded in the 2D SDF format. Then, the target protein and compounds were transformed into PDBQT format by the molecular docking Schrödinger (Maestro Version 11.1.011) software. A crystal structure docking grid box for the target protein was constructed using Schrödinger (Maestro Version 11.1.011) software. Next, docking analysis was performed, and the docking score for each pair of compounds interacting with the target protein NDUFV1 was determined. The docking score was expressed as a negative value, the lower the value, the stronger the predicted binding strength of the compound to the target protein.

### Statistical analysis

All data from the Glucose/galactose assay, SRB assay, and OCR were analyzed by GraphPad Prism 8.0 software and presented as the mean ± SD. Two-tailed Student’s t-test was used to analyze the significance of these results, and *p* values < 0.05 were considered statistically significant. The number of biological repeats was listed in each diagram.

## Results

### The establishment and validation of the dual *In Vitro* mitochondrial toxicity assay system

In this study, we established a cell-based *in vitro* mitochondrial toxicity assay system coupling a first-step screening and a second-step reverse validation. The “glucose/galactose” assay was adopted in the first-step screening, in which cells switch their energy source from glycolysis to OXPHOS. So, cells are more sensitive to mitochondrial toxicants when glucose is replaced with galactose (Swiss and Will 2011; Kamalian et al., 2015; Orlicka-Płocka et al., 2020). In this step, the human hepatocarcinoma HepG2 cell line was selected because of its super sensitivity for mitochondrial toxicity assay than other cell lines (Bugelski et al., 2000; Ma et al., 2001; Schoonen et al., 2005). In the second step, we took advantage of the human colorectal adenocarcinoma cell line HCT116 *SCO2* KO, in which the mitochondrial complex IV assembly *SCO2* gene is depleted. Compared to parental wild-type cells (HCT116 WT), HCT116 *SCO2* KO cells are deficient in respiration and resistant to cell proliferative inhibition by the mitochondrial toxicant metformin (Sung et al., 2010; Wang et al., 2017).

**FIGURE 1:**
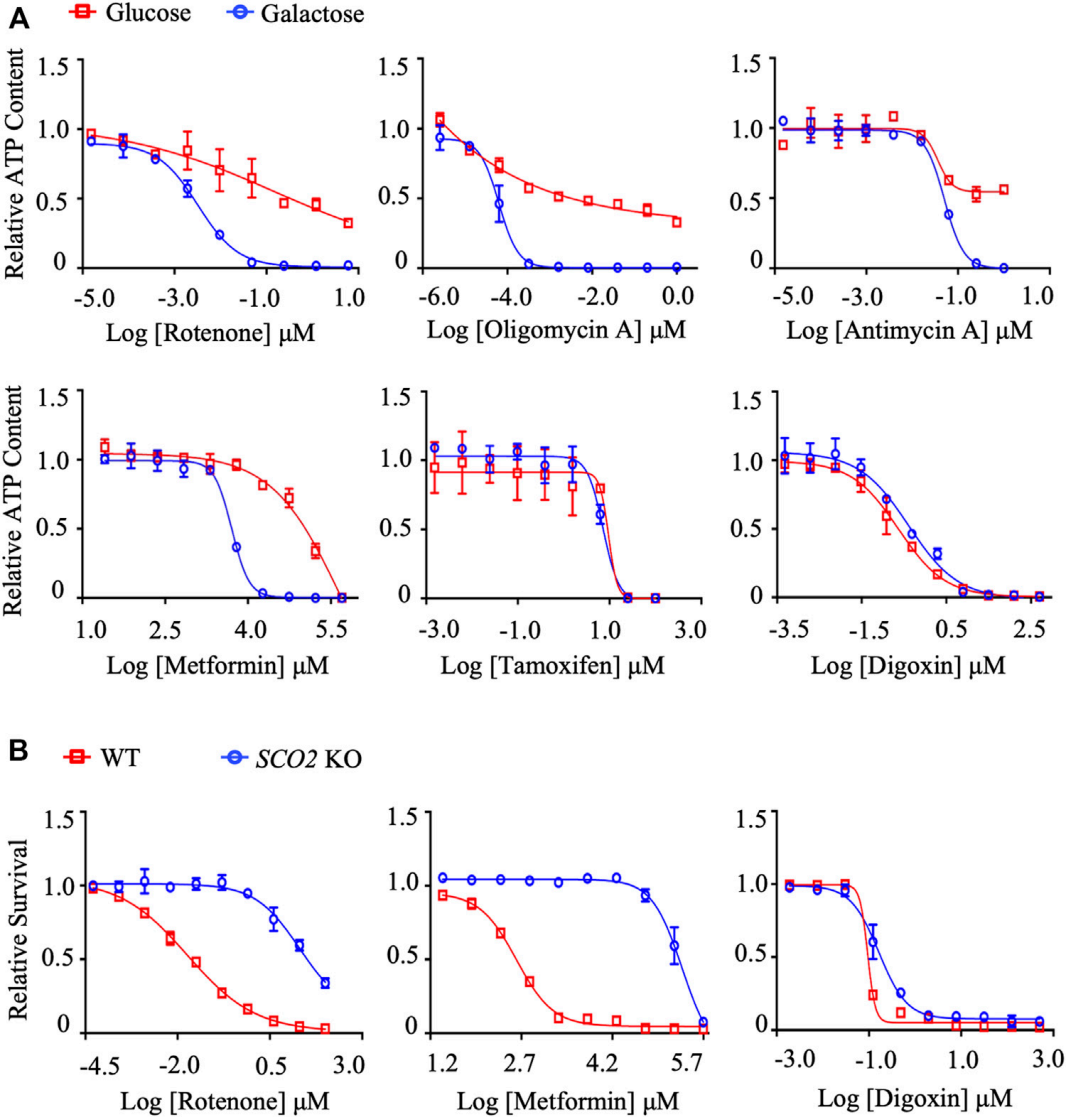
The establishment and validation of the dual *in vitro* mitochondrial toxicity assay system. **(A)** Concentration responses for high-glucose-grown (25 mM) and galactose-grown (10 mM) (blue) HepG2 cells treated with rotenone, oligomycin A, antimycin A, metformin, tamoxifen, and digoxin. After 48 h of treatment, the ATP content was assessed. **(B)** HCT-116 (WT; *SCO2* KO) cells were treated with indicated concentrations of rotenone, metformin, and digoxin for 72 h. Cell viability was assessed by the SRB assay. Average values are from three independent experiments performed in duplicate (*n* = 3). Data are shown as mean ± SD.

Mitochondrial toxicants will decrease intracellular ATP levels in galactose medium in the first assay, while are less cytotoxic in HCT116 *SCO2* KO cells in the second assay. To validate this system, we chose a well-defined set of mitochondrial toxicants, including rotenone (complex I), metformin (complex I), antimycin A (complex III), and oligomycin A (complex V), as well as cytotoxic digoxin and tamoxifen, as positive and negative control compounds, respectively. In the first assay, the IC_50_-ATP value, the concentration that results in a 50% reduction in ATP content, was calculated from dose-response curves for HepG2 cells cultured in glucose or galactose medium. As shown in Figure 1A; Supplementary Tables S3, the IC_50_-ATP values of rotenone, metformin, oligomycin A, and antimycin A were significantly reduced in galactose medium compared with that in glucose medium, while digoxin and tamoxifen did not differ significantly. According to previous publications, an IC_50_-ATP-Glu/IC_50_-ATP-Gal ratio ≥2 was defined as mitochondrial toxicity (Swiss and Will 2011; Kamalian et al., 2015; Orlicka-Płocka et al., 2020).

In the second assay, cytotoxicity was assessed by the SRB assay. Half-maximum proliferative concentrations, IC_50_ values, were calculated from dose-response curves. As expected, the positive compounds rotenone and metformin significantly inhibited cell proliferation in HCT116 WT cells compared to HCT116 *SCO2* KO cells (Figure 1B; Supplementary Tables S4). The dose-response curves and the IC50 values of the cytotoxic compound digoxin were not significantly different between the two cell lines, excluding its effect on mitochondrial function (Figure 1; Supplementary Tables S3, S4). Similarly, we defined the IC_50_-*SCO2* KO/IC_50_-WT ratio ≥2 as mitochondrial toxicity.

### Systematic evaluation of mitochondrial toxicity of TCM injections

**FIGURE 2:**
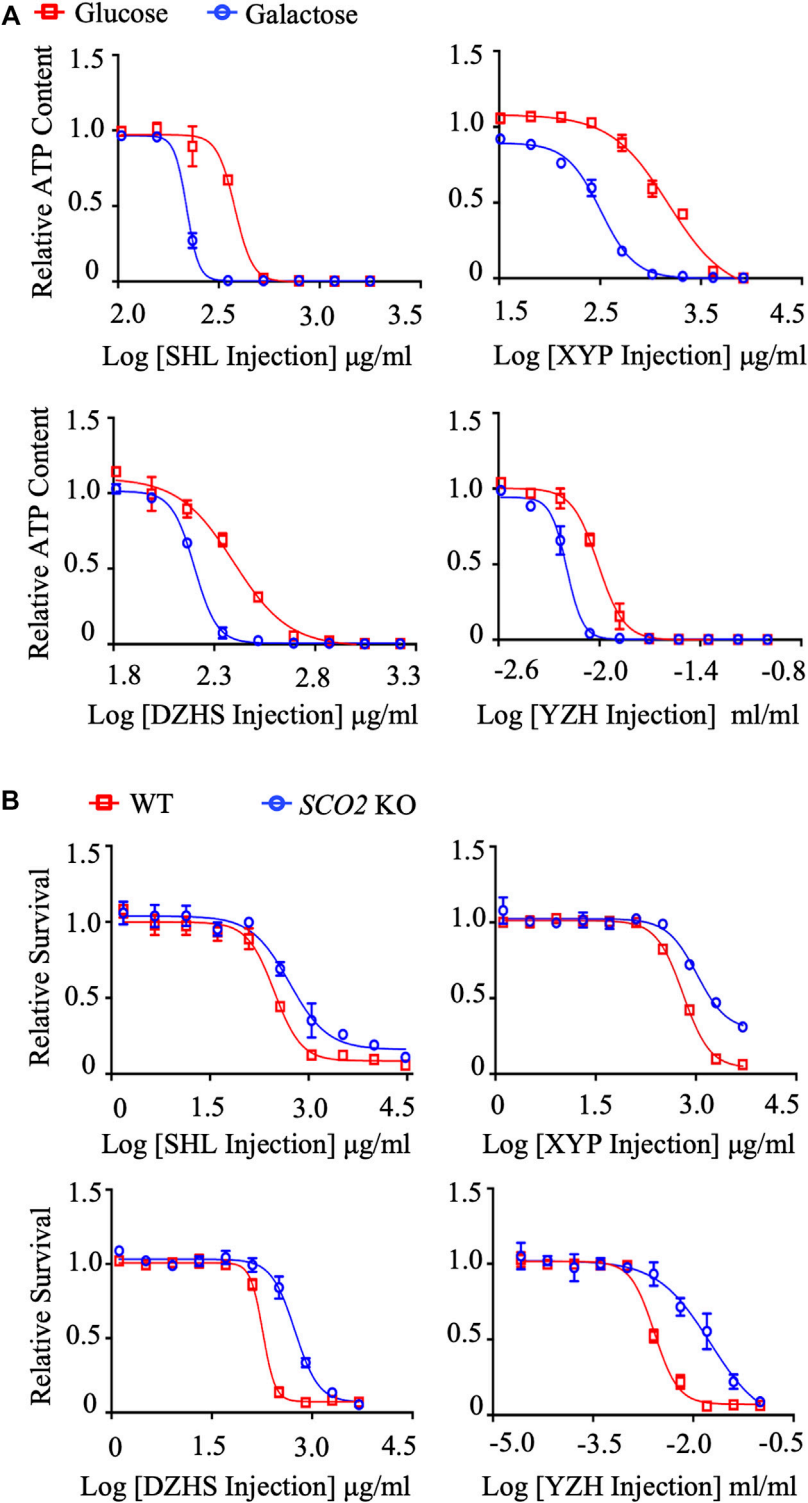
Systematic evaluation of mitochondrial toxicity of TCM injections. **(A)** Concentration responses for high-glucose-grown (25 mM) and galactose-grown (10 mM) (blue) HepG2 cells treated with XYP, DZHS, SHL and YZH injections. After 48 h of treatment, the ATP content was assessed. **(B)** HCT-116 (WT; *SCO2* KO) cells were treated with indicated concentrations of XYP, DZHS, SHL, and YZH injections for 72 h. Cell viability was assessed by the SRB assay. Average values are from three independent experiments performed in duplicate (*n* = 3). Data are shown as mean ± SD.

Using the dual *in vitro* assay approach, we systematically evaluated the mitochondrial toxicity of TCM injections we could collect. Among the 35 TCM injections tested, Xiyanping (XYP), Dengzhanhuasu (DZHS), Shuanghuanglian (SHL) and Yinzhihuang (YZH) injections exhibited the IC_50_-ATP-Glu/IC_50_-ATP-Gal ratio ≥2, ranging from 2.1061 to 5.5678, implying the potential mitochondrial toxicity (Figure 2A; Table 1). This was validated by the secondary cytotoxic assay, in which the 4 TCM injections showed greater cell proliferation inhibition in HCT116 WT cells than in HCT116 *SCO2* KO cells, and all the IC_50_-*SCO2* KO/IC_50_-WT ratios were ≥2 (Figure 2B; Supplementary Tables S2). In addition, three TCM injections, including Salvianolate, Sodium aescinate, and Shugannin, were moderately toxic to the mitochondrion in the first assay, with IC_50_-ATP-Glu/IC_50_-ATP-Gal ratios between 1 and 2 (Table 1). Two out of the three TCM injections, except Sodium aescinate, were also defined as weak mitochondrial toxicants in the second assay (Table 2). Therefore, the dual *in vitro* assays exhibited high consistency.

**TABLE 1:** The effect of TCM injections exposure on ATP depletion in HepG2 cells.

TCM injection	IC_50_-ATP	IC_50_-ATP	IC_50_-ATP	IC_50_-ATP	Mitochondrial toxicity
	Mean ± SD	Mean ± SD	Glu/Gal	Dose	Glu/Gal ≥ 2
	Glucose	Galactose	Ratio	Unit	Yes/No
Xiyanping	1541.9000	276.9333	5.5678	μg/ml	Yes
Dengzhanhuasu	323.6333	151.3333	2.1385	μg/ml	Yes
Shuanghuanglian	385.0000	191.4900	2.1796	μg/ml	Yes
Yinzhihuang	0.0124	0.0059	2.1061	ml/ml	Yes
Salvianolate	215.6000	136.4000	1.5806	μg/ml	No
Sodium aescinate	24.8200	18.5200	1.3402	μg/ml	No
Shuganning	0.0106	0.0083	1.2809	ml/ml	No
Xueshuantong	>1/100	>1/100	N/A	ml/ml	N/A
Huangqi	>1/100	>1/100	N/A	ml/ml	N/A
Reduning	>1/100	>1/100	N/A	ml/ml	N/A
Yadanziyouru	>1/100	>1/100	N/A	ml/ml	N/A
Xingnao	>1/100	>1/100	N/A	ml/ml	N/A
Danhong	>1/100	>1/100	N/A	ml/ml	N/A
Shenfu	>1/100	>1/100	N/A	ml/ml	N/A
Shenmai	>1/100	>1/100	N/A	ml/ml	N/A
Shuxuening	>100	>100	N/A	μg/ml	N/A
Yimucao	>1/100	>1/100	N/A	ml/ml	N/A
Chaihu	>1/100	>1/100	N/A	ml/ml	N/A
Lianbizhi	>100	>100	N/A	μg/ml	N/A
Shuxuetong	>1/100	>1/100	N/A	ml/ml	N/A
Ginkgo biloba extract	>100	>100	N/A	μg/ml	N/A
Tanreqing	>1/100	>1/100	N/A	ml/ml	N/A
Kushen	>1/100	>1/100	N/A	ml/ml	N/A
Shenkang	>1/100	>1/100	N/A	ml/ml	N/A
Shengmai	>1/100	>1/100	N/A	ml/ml	N/A
Kanglaite	>100	>100	N/A	μg/ml	N/A
Danshen	>100	>100	N/A	μg/ml	N/A
Yanhuning	>100	>100	N/A	μg/ml	N/A
Phloroglucinol-1	>100	>100	N/A	μg/ml	N/A
Phloroglucinol-2	>100	>100	N/A	μg/ml	N/A
Gastrodin	>100	>100	N/A	μg/ml	N/A
Yiqifumai	>100	>100	N/A	μg/ml	N/A
Astragalus polysacharin	>100	>100	N/A	μg/ml	N/A
Aidi	>1/100	>1/100	N/A	ml/ml	N/A
Shenqifuzheng	>1/100	>1/100	N/A	ml/ml	N/A

IC_50_ ATP-Glu/Gal≥2 indicates potential mitochondrial toxicity; N/A represents Non-applicable.

**TABLE 2:** The cytotoxicity of TCM injections in HCT116 (WT, *SCO2* KO) cells.

TCM injection	IC_50_-survival	IC_50_-survival	IC_50_-survival	IC_50_-survival	Mitochondrial toxicity
	Mean ± SD	Mean ± SD	(*SCO2* KO)/WT	Dose	(*SCO2* KO)/WT ≥ 2
	WT	*SCO2* KO	Ratio	Unit	Yes/No
Xiyanping	692.2000	2093.0000	3.0237	μg/ml	Yes
Dengzhanhuasu	183.1000	553.2000	3.0213	μg/ml	Yes
Shuanghuanglian	300.5000	945.8000	3.1474	μg/ml	Yes
Yinzhihuang	0.0026	0.0179	6.8177	ml/ml	Yes
Salvianolate	180.2000	257.3000	1.4279	μg/ml	No
Sodium aescinate	34.0300	21.6200	0.6353	μg/ml	No
Shuganning	0.0028	0.0032	1.1591	ml/ml	No
Xueshuantong	>1/100	>1/100	N/A	ml/ml	N/A
Huangqi	>1/100	>1/100	N/A	ml/ml	N/A
Reduning	>1/100	>1/100	N/A	ml/ml	N/A
Yadanziyouru	>1/100	>1/100	N/A	ml/ml	N/A
Xingnao	>1/100	>1/100	N/A	ml/ml	N/A
Danhong	>1/100	>1/100	N/A	ml/ml	N/A
Shenfu	>1/100	>1/100	N/A	ml/ml	N/A
Shenmai	>1/100	>1/100	N/A	ml/ml	N/A
Shuxuening	>100	>100	N/A	μg/ml	N/A
Yimucao	>1/100	>1/100	N/A	ml/ml	N/A
Chaihu	>1/100	>1/100	N/A	ml/ml	N/A
Lianbizhi	>100	>100	N/A	μg/ml	N/A
Shuxuetong	>1/100	>1/100	N/A	ml/ml	N/A
Ginkgo biloba extract	>100	>100	N/A	μg/ml	N/A
Tanreqing	>1/100	>1/100	N/A	ml/ml	N/A
Kushen	>1/100	>1/100	N/A	ml/ml	N/A
Shenkang	>1/100	>1/100	N/A	ml/ml	N/A
Shengmai	>1/100	>1/100	N/A	ml/ml	N/A
Kanglaite	>100	>100	N/A	μg/ml	N/A
Danshen	>100	>100	N/A	μg/ml	N/A
Yanhuning	>100	>100	N/A	μg/ml	N/A
Phloroglucinol-1	>100	>100	N/A	μg/ml	N/A
Phloroglucinol-2	>100	>100	N/A	μg/ml	N/A
Gastrodin	>100	>100	N/A	μg/ml	N/A
Yiqifumai	>100	>100	N/A	μg/ml	N/A
Astragalus polysacharin	>100	>100	N/A	μg/ml	N/A
Aidi	>1/100	>1/100	N/A	ml/ml	N/A
Shenqifuzheng	>1/100	>1/100	N/A	ml/ml	N/A

IC_50_-Survival- (*SCO2* KO)/WT ≥ 2 indicates potential mitochondrial toxicity; N/A represents Non-applicable.

The safety issues of the above 4 TCM injections have been widely reported. As summarized in Table 3, body as a whole-general disorders as well as skin and appendages disorders, both have allergic pathology, are the most common ADRs. The Adverse Drug Reaction Information Bulletin, issued by the National Medical Products Administration (NMPA), is the major source of information on drug safety issues in China. Due to severe allergic reactions, SHL injection was notified twice in 2005 (National Center for ADR Monitoring, 2005) and 2009 (National Center for ADR Monitoring, 2009), and XYP injection was notified once in 2012 (National Center for ADR Monitoring, 2012). In addition, DZHS injection caused liver and kidney dysfunction (Chengfeng et al., 2020; Zhang et al., 2021b). YZH and XYP injections affected the cardiovascular and nervous systems (Chen et al., 2012; Chen et al., 2016; National Medical Products Administration, 2016). As drug-induced mitochondrial toxicity can affect multiple organs, such as liver, heart, kidney, skeletal muscle, and brain (Will and Dykens 2014), we speculate that these ADRs may be related to their effects on mitochondria.

**TABLE 3:** The ADR reports of SHL, XYP, YZH, and DZHS injections.

TCM injection	Adverse drug reactions (ADRs)	References
Shuanghuangliuan (SHL)	1) Body as a whole-general disorders (anaphylactic shock, anaphylactic reaction, high fever, chills)	Chen et al. (2012), Feng et al. (2018), Gao et al. (2018), Gu et al. (2018), Han et al. (2018), Li et al. (2018), National Center for ADR Monitoring, (2005), National Center for ADR Monitoring, (2009), Tan et al. (2019), Wang et al. (2010a), Wang et al. (2010b), Xiao et al. (2020), Yi et al. (2012), Zhang et al. (2018)
	2) Respiratory disorders (Dyspnea, tachypnea, laryngeal edema, bronchospasm)	
	3) Skin and appendages disorders (Eruptive drug eruption, vascular neurotic edema, exfoliative dermatitis, henoch purpura, severe erythema multiforme)	
	4) Other impairments (liver function impairment, nephrotoxicity, proarrhythmic risk, decreased blood pressure, visual abnormalities, auditory abnormalities, convulsions and coma)	
Xiyanping (XYP)	1) Body as a whole-general disorders (Anaphylactic reaction, anaphylactic shock, chills, fever, palpitations, diarrhea, Dizziness headache)	Chen et al. (2018), Chen et al. (2016), He and Wei (2017), Li et al. (2018), National Center for ADR Monitoring, (2012), Yang et al. (2019), Zhao et al. (2021a)
	2) Respiratory disorders (Dyspnoea, cough)	
	3) Skin and appendages disorders (Rash, urticaria accompanied by itching)	
	4) Cardiovascular disorders (Cyanosis)	
	5) Other impairments (Persistent congestion and swelling of both eyes)	
Yinzhihuang (YZH)	1) Body as a whole-general disorders (anaphylactic shock and even fatal, fever with systemic damage)	National Medical Products Administration, (2016), Chen et al. (2012), Feng et al. (2022), Wang et al. (2010b), Xie and Ye (2016)
	2) Skin and accessory damage (Pruritus and rash)	
	3) Other impairments (increased hemolysis, blood system damage, medication local damage proarrhythmic risk, digestive system damage, nervous system damage)	
Dengzhanhuasu (DZHS)	1) Body as a whole-general disorders (allergic reactions, anaphylactic shock, palpitation, dizziness, headache, chest tightness, high fever, abdominal distension and diarrhea, convulsion of limbs, nausea and vomiting)	Chengfeng et al. (2020), Guo and Li (2012), Li et al. (2019), Tan et al. (2019), Zhang et al. (2021a), Zhang et al. (2021b), Zheng (2019)
	2) Skin and appendages disorders (skin pruritus, flushes, chronic urticaria)	
	3) Other impairments (Renal dysfunction, liver dysfunction)	

### Identification of mitochondrial toxic ingredients by molecular docking

**FIGURE 3:**
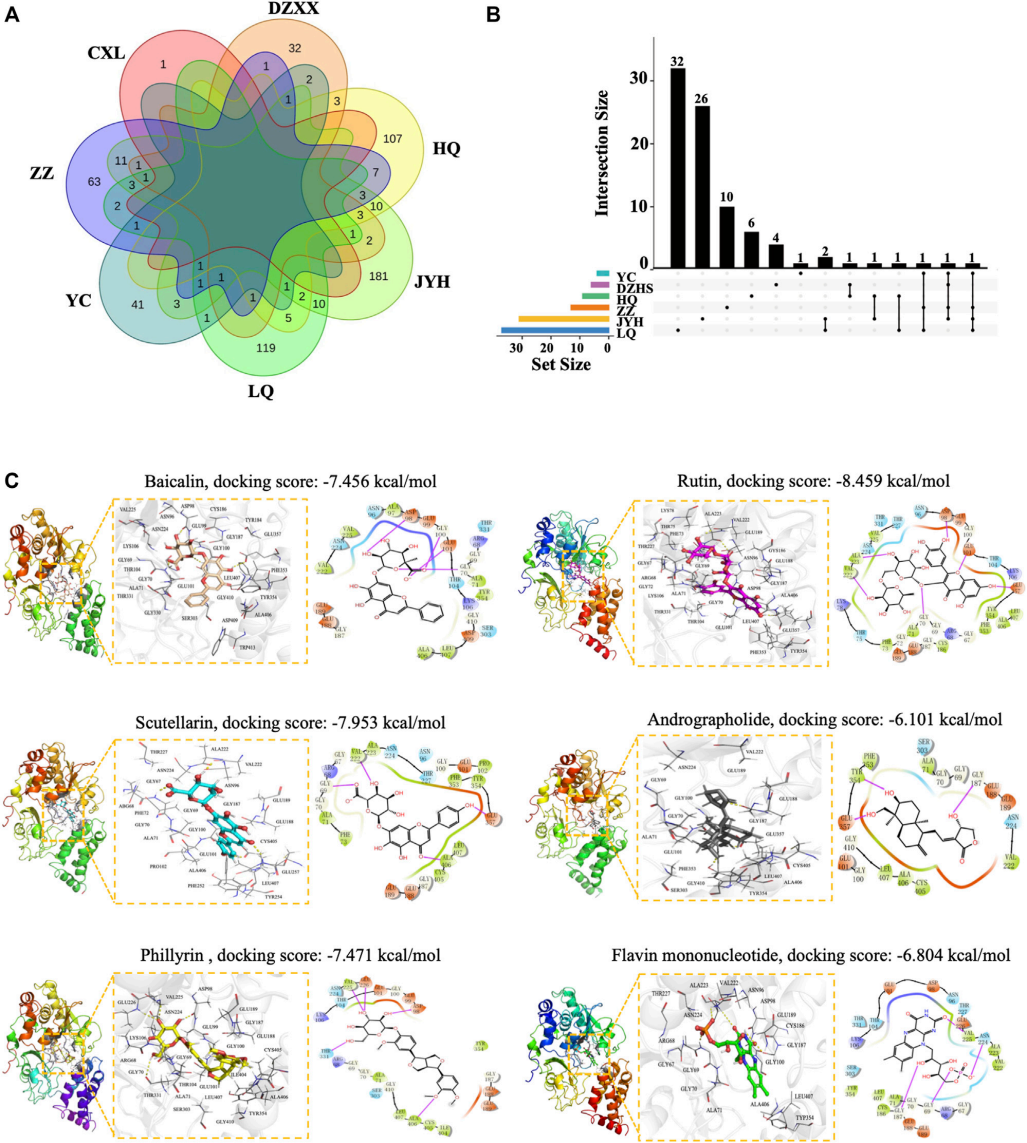
Identification of mitochondrial toxic ingredients by molecular docking. **(A)** Venn diagram of ingredients from Huangqin (HQ), Jinyinhua (JYH), Lianqiao (LQ), Yinchen (YC), Zhizi Dengzhanxixin (DZXX), and Chuanxinlian (CXL). **(B)** UpSetR plot of ingredients with a docking score below −6.804. **(C)** Molecular modes of the binding of compounds with NDUFV1 complex shown as 3D and 2D diagrams. The hydrogen bond is indicated by a yellow dotted line (left) and the purple arrows represent hydrogen bond interactions between molecules. Baicalin, scutellarin, rutin, phillyrin, andrographolide, and the natural ligand flavin mononucleotide are shown in orange, cyan, yellow, magenta, gray, and green sticks respectively, and key residues are shown as a line in white (left).

It is of great significance to identify mitochondrial toxic ingredients from the 4 TCM injections. On the one hand, it can be inferred that other TCM products containing the same ingredients may have similar concerns. On the other hand, by minimizing the content of the harmful components, the safety of related products can be improved. To this end, we first retrieved all known ingredients of the 4 TCM injections from the TCMSP database (https://tcmsp-e.com/). A total of 622 ingredients from the seven common herbs were found after eliminating the overlaps, including 143 in *Scutellariae Radix,* 53 in *Artemisiae Scopariae Herba,* 49 in *Erigeron Breviscapus,* 236 in *Lonicerae Japonicae Flos,* 150 in *Forsythiae Fructus,* 98 in *Gardeniae Fructus,* and 1 in *Andrographis Herba* (Figure 3A). Next, we employed molecular docking to identify potential mitochondrial toxic compounds. Complex I (NADH: ubiquinone oxidoreductase), the largest enzyme of the mitochondrial respiratory chain, is the most common binding target of mitochondrial toxic drugs (Imaizumi et al., 2015; Murai and Miyoshi 2016). NADH dehydrogenase [ubiquinone] flavoprotein 1, mitochondrial (NDUFV1) is the core subunit of complex I, and its binding with compounds results in defects in the electron transfer chain (Zhu et al., 2016). In addition, by empirical judgment and Sitemap (the module of Schrödinger (Maestro Version 11.1.011) software) analysis, NDUFV1 is the best protein-ligand binding pocket in complex I. Therefore, we took NDUFV1 as an example to perform molecular docking with 622 compounds from 4 TCM injections.

After eliminating the overlaps, a total of 87 compounds showed a strong binding ability to NDUFV1 with a docking score lower than −6.804, the value for the natural ligand flavin mononucleotide. Among the 87 compounds, there are 9 in *Scutellariae Radix,* 4 in *Artemisiae Scopariae Herba,* 6 in *Erigeron Breviscapus,* 31 in *Lonicerae Japonicae Flos,* 37 in *Forsythiae Fructus,* and 13 in *Gardeniae Fructus* (Figure 3B; Supplementary Tables S5). Baicalin and phillyrin in SHL and YZH and scutellarin in DZHS are quality control ingredients in the “Chinese Pharmacopeia 2020 Edition” (Commission 2020). In addition, baicalin, rutin, and phillyrin have been reported to be associated with various ADRs (Table 4). XYP injection is a mixture of andrographolide sulfate, which is a water-soluble medicament prepared from andrographolide extracted from *Andrographis Herba* through sulfonating reaction (Chong et al., 2013; Zheng et al., 2016). Therefore, we further analyzed the binding modes of interaction between these compounds and the target protein. From the 3D crystal structure of the small molecule-NDUFV1 complex, it can be seen that the distance between the amino acid residue and the small molecule was less than five Å (Figure 3C). The docking scores of scutellarin, rutin, phillyrin, and baicalin were −7.456 kcal/mol, −7.953 kcal/mol, −7.471 kcal/mol, and −8.459 kcal/mol, respectively (Figure 3C). So, these compounds can bind strongly to NDUFV1, suggesting their potential mitochondrial toxicity. In line with our findings, it has been recently reported that anthracene-9,10-dione and phthalaldehyde, a series of flavonoid derivative substructures, including baicalin, scutellarin, and rutin, were mitochondrial toxic identified by machine learning (Zhao P. et al., 2021).

**TABLE 4:** The toxicity reports of the ingredients from TCM injections.

Compound	Toxicity reports	References
Baicalin	1) Body as a whole-general disorders	Cai et al. (2017), Deng et al. (2017), Gao et al. (2014), Shii et al. (2020), Wang et al. (2020), Zhang et al. (2017), Zhang et al. (2012), Zhang et al. (2021d), Zhang et al. (2021c), Zhao et al. (2021b)
	2) Liver dysfunction (liver fibrosis)	
	3) Kidney dysfunction (renal fibrosis)	
	4) Other impairments (interstitial pneumonia, exert weak decomposition toxicity, Embryo toxicity)	
Rutin	1) Body as a whole-general disorders (anaphylactoid reaction; headache, rashes, muscle stiffness)	Sanz-Serrano et al. (2021), Wilson (2017), Zhang et al. (2017), Zhao et al. (2021b)
	2) Cardiovascular disorders (slow or fast heartbeat)	
	3) Nervous system disorder (blurred vision, nervousness)	
	4) Other impairments (genotoxicity, producing frameshift mutations)	
Phillyrin	1) Low probability of genetic toxicitylow sub-chronic toxicity	Han et al. (2021), Han et al. (2017)
Scutellarin	1) No adverse reactions have been reported	Melamed-Gal et al. (2018), Wu et al. (2021), Zhao et al. (2021b)

### *In Vitro* validation of mitochondrial toxicity of identified TCM ingredients

**FIGURE 4:**
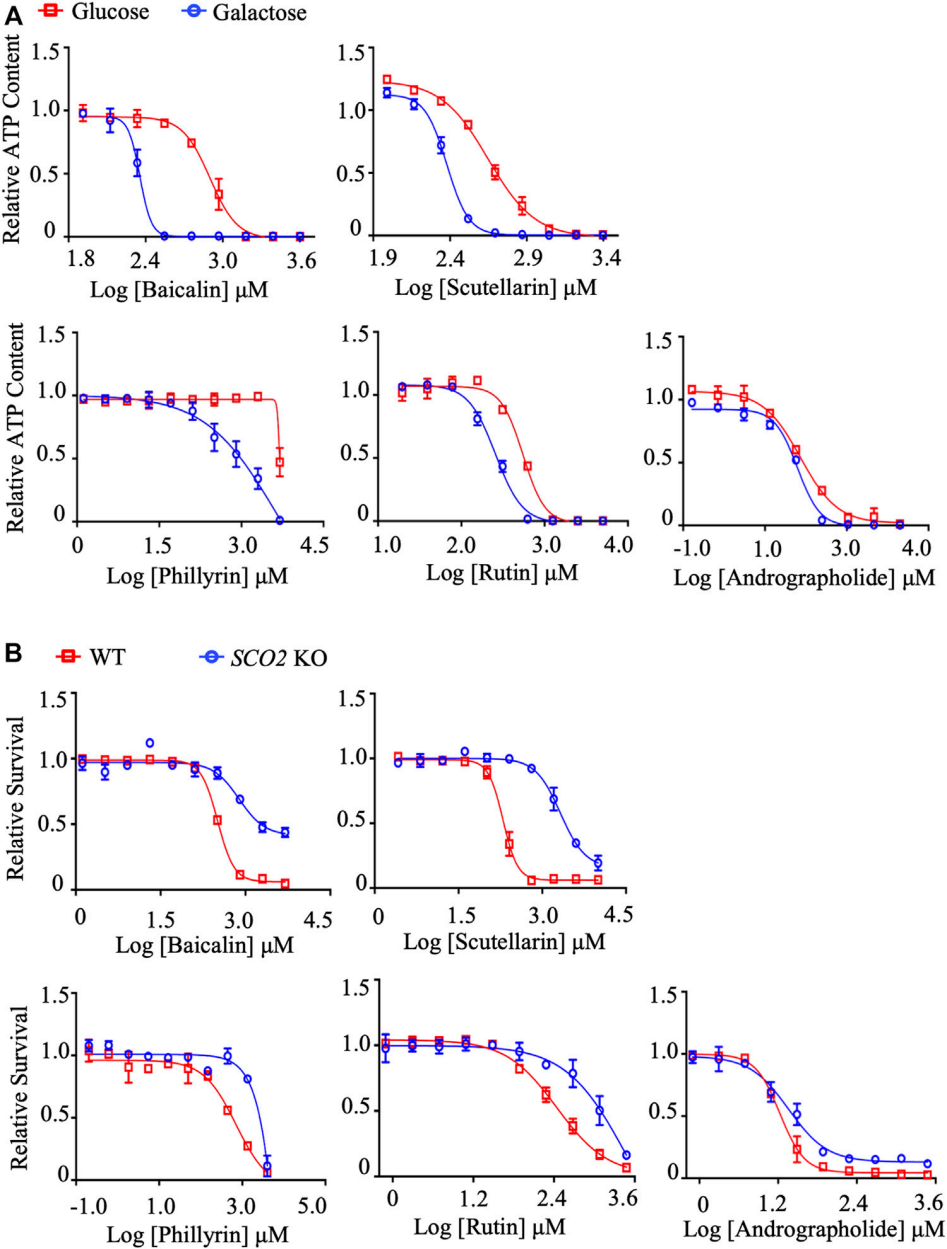
*In vitro* validation of mitochondrial toxicity of identified TCM ingredients. **(A)** Concentration responses for high-glucose-grown (25 mM) and galactose-grown (10 mM) (blue) HepG2 cells treated with baicalin, scutellarin, rutin, phillyrin, and andrographolide. After 48 h of treatment, the ATP content was assessed. **(B)** HCT-116 (WT; *SCO2* KO) cells were treated with indicated concentrations of baicalin, scutellarin, rutin, phillyrin, and andrographolide for 72 h. Cell viability was assessed by the SRB assay. Average values are from three independent experiments performed in duplicate (*n* = 3). Data are shown as mean ± SD.

To validate the findings of molecular docking, these compounds were subjected to the dual *in vitro* mitochondrial toxicity assay. As expected, all compounds showed an IC_50_-ATP-Glu/IC_50_-ATP-Gal ratio ≥2 (Figure 4A **;** Table 5) and an IC_50_-*SCO2* KO/IC_50_-WT ratio ≥2 (Figure 4B; Table 6), which demonstrated to be mitochondrial toxic. As summarized in Table 4, baicalin and rutin are the main components in SHL injection causing the anaphylactoid reaction (Zhang et al., 2017; Wang et al., 2020). Baicalin, also present in YZH injection, has been reported to induce kidney damage and renal fibrosis (Cai et al., 2017). Rutin, a plant pigment contained in SHL and YZH injections, is generally safe, but overdose can cause cardiovascular and neurological disorders (Wilson 2017). Although phillyrin in SHL injection and scutellarin in DZHS injection were identified as potential mitochondrial toxicants by molecular docking analysis (Figure 3C) and our dual *in vitro* approach (Figures 4A,B), there are few reports of their ADRs (Table 4).

**TABLE 5:** The effect of ingredients from TCM injections exposure on ATP depletion in HepG2 cells.

Compound	IC_50_-ATP	IC_50_-ATP	IC_50_-ATP	IC_50_-ATP	Mitochondrial toxicity
	Mean ± SD	Mean ± SD	Glu/Gal	Dose	Glu/Gal ≥ 2
	Glucose	Galactose	Ratio	Unit	Yes/No
Rutin	549.4000	256.6000	2.1411	μM	Yes
Baicalin	1203.5000	432.4000	2.7833	μM	Yes
Scutellarin	521.7000	252.2000	2.0686	μM	Yes
Phillyrin	3757.0000	769.2000	4.8843	μM	Yes
Andrographolide	53.9500	41.2433	1.3081	μM	No

IC_50_ ATP-Glu/Gal≥2 indicates potential mitochondrial toxicity.

**TABLE 6:** The cytotoxicity of ingredients from TCM injections in HCT116 (WT, *SCO2* KO) cells.

Compound	IC_50_-survival	IC_50_-survival	IC_50_-survival	IC_50_-survival	Mitochondrial toxicity
	Mean ± SD	Mean ± SD	(*SCO2* KO)/WT	Dose	(*SCO2* KO)/WT ≥ 2
	WT	*SCO2* KO	Ratio	Unit	Yes/No
Rutin	276.3000	2966.0000	10.7347	μM	Yes
Baicalin	322.4000	770.0000	2.3883	μM	Yes
Scutellarin	197.6000	2104.0000	10.6478	μM	Yes
Phillyrin	540.3000	2105.0000	3.8960	μM	Yes
Andrographolide	16.8600	22.7800	1.3511	μM	No

IC_50_-Survival-(*SCO*2 KO)/WT ≥ 2 indicates potential mitochondrial toxicity.

XYP injection is a mixture of andrographolide sulfate made from andrographolide extracted from *Andrographis Herba* through sulfonation reaction to increase water solubility, bioavailability, and stability (Loureiro Damasceno et al., 2022; Zou et al., 2022). Andrographolide is the main component of XYP injection. However, its docking score with NDUFV1 was not significant (−6.101 kcal/mol) (Figure 3C), which led us to speculate that its mitochondrial toxicity may not be due to its binding to complex I. To our surprise, neither the IC_50_-ATP-Glu/IC_50_-ATP-Gal ratio (Figure 4A; Table 5) nor the IC_50_-*SCO2* KO/IC_50_-WT ratio (Figure 4B; Table 6) of andrographolide was ≥2, indicating lower mitochondrial toxicity compared with XYP injection (Figure 2; Table 1, 2). This discrepancy indicates that the mitochondrial toxicity of XYP injection may come from uncharacterized components during the extraction process or during the sulfonation reaction, which needs further investigation.

### Effects of mitochondrial toxic TCM injections and ingredients on the respiration of HepG2 cells

**FIGURE 5:**
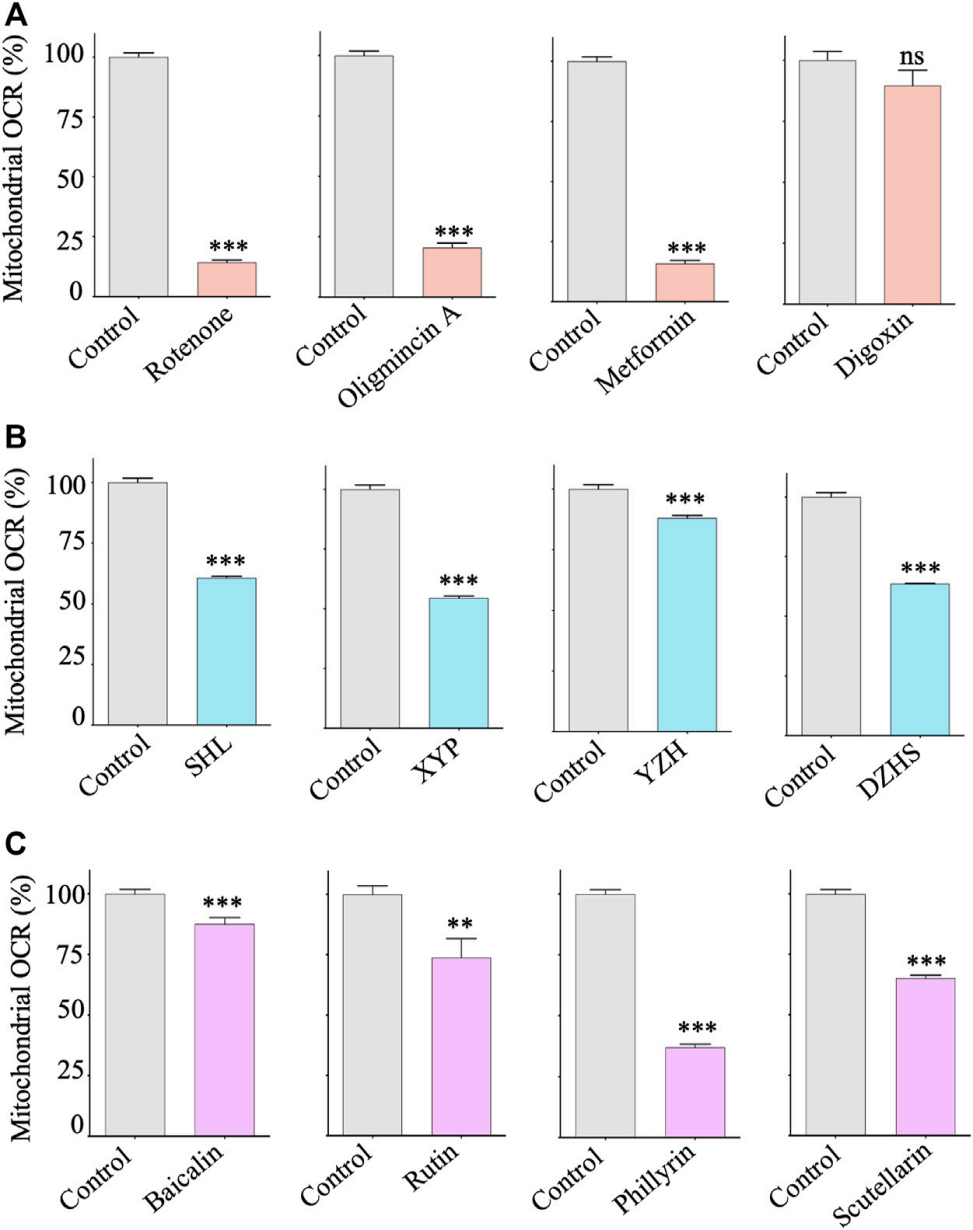
Effects of mitochondrial toxic TCM injections and ingredients on the respiration of HepG2 cells. HepG2 cells were treated with control compounds, TCM injections and ingredients with indicated concentrations for 24 h. The mitochondrial respiration rate (OCR) was assessed by the Instech Fiber Optic Oxygen Monitor System. **(A)** Control compounds: rotenone (2 μM), oligomycin A (2 μM), metformin (10 mM), and digoxin (2 μM). **(B)** TCM injections: SHL (200 μg/ml), XYP (500 μg/ml), YZH (0.01 ml/ml), and DZHS (200 μg/ml). **(C)** TCM ingredients: baicalin (200 μM), rutin (200 μM), phillyrin (500 μM), and scutellarin (200 μM). Average values are from three independent experiments performed in duplicate (*n* = 3). Data are analyzed by two-tailed Student’s t-test and presented as mean ± SD. (*p < 0.05, **p < 0.01, *** *p* <0.001; ns, nonsignificant).

Measurement of oxygen consumption rate (OCR) has long been used as the gold standard for evaluating drug-induced mitochondrial toxicity (Meyer et al., 2018). To validate the identified mitochondrial toxic TCM injections and ingredients, we performed OCR assays in HepG2 cells using a fluorescence lifetime micro-oxygen monitoring system. As expected, positive mitochondrial toxicants, such as rotenone, oligomycin A and metformin, significantly inhibited OCR levels (Figure 5A). Similar effects were observed for the identified TCM injections and their ingredients (Figures 5B,C). Taken together, these results suggest that XYP, DZHS, SHL, and YZH injections have potential mitochondrial toxicity, which may be related to their ADRs. Their adverse effects on mitochondria are due at least in part to the toxic components baicalin, rutin, scutellarin, and phillyrin.

## Discussion

Although TCM injections have benefited China’s health system for decades, concerns about their safety have grown in recent years. The China National Medical Products Administration (NMPA) thus put forward mandatory requirements for post-marketing re-evaluation of TCM injections in 2018 (Drug Evaluation Center 2018). Drug-induced mitochondrial dysfunctions are one of the major underlying mechanisms of ADRs, and related detection methods have been widely used in the modern pharmaceutical industry but rarely in TCM products. Wang et al. (2015) carried out a pilot mitochondrial toxicity study using high content microscopy of four TCM injections, including Danhong, Xiangdan, Mailuoning, and Fufangkushen. They found that mitochondrial dysfunction may be related to the hepatotoxicity of Fufangkushen injection. However, the requirements for expensive equipment limit the application of this assay. In this study, we adopted the more affordable “glucose/galactose” assay for mitotoxicants screening, combined with the SRB cytotoxicity assay on a cell line deficient in mitochondrial respiration for validation. The dual *in vitro* mitochondrial toxicity assay system provides additional accuracy for mitochondrial toxicity prediction compared to the one-step “glucose/galactose” assay. Furthermore, the system is suitable for high-throughput detection.

It should be noted that there are other well-established methods and assays to study drug-induced mitochondrial toxicity that can be assessed in isolated mitochondria, cells, and tissue. As summarized by Mihajlovic *et al.* these methods include mitochondrial membrane potential (MMP), mitochondrial permeability transition pore (MPTP) opening, mitochondrial NADPH and NADH, mitochondrial ROS, mtDNA copy number, and mitochondrial morphology, size, and number (Mihajlovic and Vinken 2022). In addition, *in silico* prediction approaches have been recently developed (Rana et al., 2019; Hemmerich et al., 2020; Troger et al., 2020). However, above methods also have some disadvantages, including high toxicity, low sensitivity, poor specificity, and high cost of some probes when used alone. Compared with these methods, the advantages of the dual *in vitro* approach were inexpensive, consistent, reproducible, high-throughput and additional accuracy for mitochondrial toxicity prediction. And the disadvantages of the dual *in vitro* approach were the lack of the overall response, such as live kidney function and myocardial enzymes, etc. Therefore, the selection and combination of these methods can provide more convenient and robust strategies for mitochondrial toxicity assessment and can contribute to the TCM safety research society.

Mechanistically, one of the most prominent causes of mitochondrial toxicity of compounds is the interaction with respiratory chain complexes. For the first time, we carried out molecular docking studies to screen mitochondrial toxic compounds from TCM products. However, our attempts were limited to NDUFV1, the core subunit of complex I. To comprehensively predict the mitochondrial toxic ingredients of TCM injections, the targets of other complexes should also be considered. The 3D structures of SDHA subunit in complex II (Sun et al., 2005), UQCRFS1 subunit in complex III (Xia et al., 2013), NDUFA4 subunit in complex IV (Balsa et al., 2012), and α (ATP5A1) subunit in complex V (Abbas et al., 2020) have been solved and can be used for molecular docking analysis of TCM injection compounds. Meanwhile, our molecular docking analysis revealed a large number of TCM compounds with a high binding ability to mitochondria, providing a rich database for machine learning. Although the TCMSP database is very professional and reliable, in future studies, we should consider combining with other techniques, such as chromatography tandem mass spectrometry, to predict the mitochondrial toxicity of TCM ingredients.

Currently, after Lianbizhi withdraws from the market in 2022, there are 133 TCM injections approved by the NMPA (http://www.nmpa.gov.cn/WS04/CL2042/). Although we could not collect all of them due to the temporary suspension of sales for some TCM injections, 35 is an acceptable representative number. Therefore, we systematically evaluated the mitochondrial toxicity of TCM injections for the first time. The percentage of TCM injections with mitochondrial toxicity, 4 out of 35, is relatively high, implying all the other TCM injections need to be screened immediately. It is also suggested the necessity of mitochondrial toxicity evaluation for other dosage forms of TCM.

On the one hand, mitochondrial toxicants are intrinsic constituents of TCM products, such as those mentioned in the introduction section, as well as scutellarin, baicalin, rutin, and phillyrin found in this study. On the other hand, they may also come from environmental containments introduced during the cultivation and processing of TCM products, such as air pollutants, pesticides, and heavy metals known to be toxic to mitochondria (Roubicek and Souza-Pinto, 2017). This suggests that mitochondrial toxicity assessment should be considered throughout the entire process of TCM production, and re-emphasizes the importance of Good Agricultural Practice (GAP) and Good Manufacturing Practice (GMP) to the TCM industry.

The identified mitochondrial toxic compounds exist in a variety of herbal medicines. For example, according to the HERB database (http://herb.ac.cn/), Chinese herbal medicines such as *Radix Scutellariae, Radix Bupleuri, Flos Carthami,* and *Radix Salviae liguliobae* contain baicalin and rutin. Therefore, attention should be paid to other TCM products containing these mitochondrial toxic compounds. Although Chaihu, Danhong, and Danshen injections contain baicalin and rutin, and Tanreqing injection contains baicalin, rutin, and phillyrin, they did not exhibit strong mitochondrial toxicity in our dual *in vitro* system. This may be due to the relatively low concentrations of these compounds in these TCM injections.

Anaphylactic shock and anaphylactoid reaction are the most common ADRs of TCM injections and have been reported in the 4 mitochondrial toxic TCM injections identified in this study (Li et al., 2019). Like other mitochondrial toxicants, mitochondria damage induced by TCM injections results in the release of damage-associated molecular patterns (mtDAMPs), including ATP, ROS, cardiolipin, mitochondrial DNA (mtDNA), and formyl peptides. Once leak into the cytosol, mtDAMPs can potentiate inflammatory and type I interferon responses through the engagement of innate sensors, such as TLR9, cGAS, NLRs, and FPRs (West, 2017).

Certain populations are more susceptible to mitochondrial toxicants. Primary mitochondrial disease (PMD) refers to a group of metabolic disorders due to mitochondrial dysfunction caused by germline mutations in mtDNA or nuclear DNA (Niyazov et al., 2016). PMDs, such as Leigh syndrome, Kearns-Sayre syndrome, Alpers-Huttenlocher syndrome, mitochondrial encephalomyopathy with lactic acidosis and stroke-like episodes (MELAS) syndrome, and ataxia neuropathy syndrome, share similar mechanisms and symptoms with mitochondrial toxicants induced ADRs. So, the latter play important roles in the penetrance and pathogenesis of PMD patients (Chan, 2017). The “mitochondrial theory of aging” proposes that mitochondrial function declines with age and has a casualty relationship with age-related diseases such as cardiovascular disease, Parkinson’s, Alzheimer’s and Huntington’s disease, hearing loss, sarcopenia, inflammation, and cancer (Bratic and Larsson 2013). Mitochondrial toxicants can increase the frequency and severity of these diseases (Will et al., 2019). Therefore, particular attention should be paid to the use of TCM products among the elderly, who are the main consumer population, usually on a long-term basis (De Souza Silva et al., 2014).

## Conclusion

In summary, we have established a dual *in vitro* mitochondrial toxicity screen system by combing the “glucose/galactose” assay in HepG2 cells with the cytotoxicity assay in mitochondrial respiration deficient cells. Using this system, we systematically evaluated mitochondrial toxicity in TCM injections for the first time. The method is worth applying to the safety evaluation of more TCM products.

## Data Availability

The raw data supporting the conclusions of this article will be made available by the authors, without undue reservation.
