# Supplementary material for: Mitochondrial toxicity evaluation of traditional Chinese medicine injections with a dual in vitro approach
Source: Front Pharmacol. 2022 Nov 2;13:1039235. doi: 10.3389/fphar.2022.1039235 (PMC9667049; doi:10.3389/fphar.2022.1039235)
Supplement: Supplementary file 1 [file Table1.DOCX]

| **TCM Injection** | **Drug approval Number** | **Batch Number** | **Commercial Supplier** |
| --- | --- | --- | --- |
| Xiyanping | Z20026249 | 2017041403 | Jiangxi Qingfeng Pharmaceutical Co., Ltd |
| Dengzhanhuasu | Z53020667 | 20170221-2 | Kunming Longjin Pharmaceutical Co., Ltd |
| Shuanghuanglian | Z20043425 | 1611015 | Harbin Pharmaceutical Group |
| Yinzhihuang | Z13020772 | 170613D1 | Shenwei Pharmaceutical Co., Ltd |
| Xueshuantong | Z45021769 | 17011609 | Guangxi Wuzhou Pharmaceutical (Group) Co., Ltd |
| Huangqi | Z23020782 | A03161201 | Heilongjiang Zhenbaodao Pharmaceutical Co., Ltd |
| Reduning | Z20050217 | 170520 | Jiangsu Kanyuan Pharmaceutical Co., Ltd |
| Yadanziyouru | Z44021325 | 170101 | Guangzhou Baiyunshan Mingxing Pharmaceutical Co., Ltd |
| Xingnao | Z53021638 | 1703042 | Dali Pharmaceutical Co., Ltd |
| Danhong | Z20026866 | 16121032 | Shandong Danhong Pharmaceutical Co., Ltd |
| Shenfu | Z51020664 | 17010201006 | China Resources Sanjiu Pharmaceutical Co., Ltd |
| Shenmai | Z51020552 | 16120102001 | China Resources Sanjiu Pharmaceutical Co., Ltd |
| Shuxuening | Z13020795 | 16071202 | Shenwei Pharmaceutical Co., Ltd |
| Yimucao | Z51021448 | 170401 | Chengdu First Pharmaceutical Co., Ltd |
| Chaihu | Z14021017 | 1611171 | Shanxi Jinxin Double Crane Pharmaceutical Co., Ltd |
| Shuganning | Z20025660 | 20170718 | Guizhou Ruihe Pharmaceutical Co., Ltd |
| Lianbizhi | Z32021034 | 170503 | Wuxi Jimin Trust Shanhe Pharmaceutical Co., Ltd |
| Shuxuetong | Z20010100 | 160718 | Mudanjiang Youbo Pharmaceutical Co., Ltd |
| Ginkgo biloba extract | H20070226 | 18870225 | Yuekang Pharmaceutical Group Co., Ltd |
| Tanreqing | Z20030054 | 1702113 | Shanghai Kaibao Pharmaceutical Co., Ltd |
| Kushen | Z14021231 | 20161120 | Shanxi Zhendong Pharmaceutical Co., Ltd |
| Shenkang | Z20040110 | 201706043 | Xian Shiji Shengkang Pharmaceutical Co., Ltd |
| Shengmai | Z51021882 | 16121205002 | China Resources Sanjiu Pharmaceutical Co., Ltd |
| Kanglaite | Z10970091 | 1702226-2 | Zhejiang Kanglei Pharmaceutical Co., Ltd |
| Salvianolate | Z20050249 | 17031621 | Shanghai Green Valley Pharmaceutical Co., Ltd |
| Danshen | Z23021510 | 1611730 | Harbin Pharmaceutical Group |
| Yanhuning | H20046051 | 20170403H | Wuhan Changlianliafu Pharmaceutical Co., Ltd |
| Sodium aescinate | H20067083 | 160719B | Wuhan Aimin Pharmaceutical Co., Ltd |
| Phloroglucinol-1 | H20060385 | 17080302 | Hubei Noon Time Pharmaceutical Co., Ltd |
| Phloroglucinol-2 | H20046766 | 30170610 | Nanjing Hengsheng Pharmaceutica Co., Ltd |
| Gastrodin | H20013046 | 17AX10-231 | Kunming Pharmaceutical Group Co., Ltd |
| Yiqifumai | Z20060463 | 20190405 | Tianjin Tasly Zhijiao Pharmaceutical Co., Ltd |
| Astragalus polysacharin | Z20040086 | 20171203 | Tianjin Saino Pharmaceutical Co., Ltd |
| Aidi | Z52020236 | 20181215 | Guizhou Yibai Pharmaceutical Co., Ltd |
| Shenqifuzheng | Z19990065 | 20190114 | Lizhu Group Limin pharmaceutical Co., Ltd |

**Supplementary Table S1.** All TCM injections were respectively purchased from indicated commercial suppliers.
