# Supplementary material for: Mitochondrial toxicity evaluation of traditional Chinese medicine injections with a dual in vitro approach
Source: Front Pharmacol. 2022 Nov 2;13:1039235. doi: 10.3389/fphar.2022.1039235 (PMC9667049; doi:10.3389/fphar.2022.1039235)
Supplement: Supplementary file 2 [file Table2.DOCX]

**Supplementary Table S2.** All control compounds and ingredients from TCM injections were respectively purchased from indicated commercial suppliers.

| **Compound** | **Commercial Supplier** |
| --- | --- |
| Rotenone | Sigma Aldrich (St. Louis, MO, USA) |
| Oligomycin A | Sigma Aldrich (St. Louis, MO, USA) |
| Antimycin A | Sigma Aldrich (St. Louis, MO, USA) |
| Metformin | Sigma Aldrich (St. Louis, MO, USA) |
| Tamoxifen | Sigma Aldrich (St. Louis, MO, USA) |
| Digoxin | Alfa (Shanghai, China) |
| Rutin | Dalian Meilun Biotechnology Co., Ltd (Dalian, China) |
| Andrographolide | Dalian Meilun Biotechnology Co., Ltd (Dalian, China) |
| Baicalin | Dalian Meilun Biotechnology Co., Ltd (Dalian, China) |
| Scutellarin | Dalian Meilun Biotechnology Co., Ltd (Dalian, China) |
| Philyrin | Dalian Meilun Biotechnology Co., Ltd (Dalian, China) |
