# Supplementary material for: Mitochondrial toxicity evaluation of traditional Chinese medicine injections with a dual in vitro approach
Source: Front Pharmacol. 2022 Nov 2;13:1039235. doi: 10.3389/fphar.2022.1039235 (PMC9667049; doi:10.3389/fphar.2022.1039235)
Supplement: Supplementary file 3 [file Table5.DOCX]

**Supplementary Table S5.** Prediction of mitochondrial toxicity by molecular docking of ingredients in identified TCM injections with the subunit NDUFV1 (Docking score < -6.804).

| **Herb Name** | **Mol ID** | **Molecule Name** | **Docking Score (kcal/mol)** |
| --- | --- | --- | --- |
| *Scutellariae Radix* | MOL007792 | Isomartynoside | -8.69 |
| *Scutellariae Radix* | MOL002912 | Dihydrobaicalin | -8.19 |
| *Scutellariae Radix* | MOL002931 | Scutellarin | -7.95 |
| *Scutellariae Radix* | MOL002921 | Darendroside B | -7.92 |
| *Scutellariae Radix* | MOL002935 | Baicalin | -7.46 |
| *Scutellariae Radix* | MOL013068 | Oroxindin | -7.07 |
| *Scutellariae Radix* | MOL006370 | 5-o-caffeoylquinic acid | -6.96 |
| *Scutellariae Radix* | MOL002923 | Darendoside B | -6.87 |
| *Scutellariae Radix* | MOL000007 | Cosmetin | -6.83 |
| *Artemisiae Scopariae Herba* | MOL000415 | Rutin | -8.43 |
| *Artemisiae Scopariae Herba* | MOL000437 | Hirsutrin | -7.51 |
| *Artemisiae Scopariae Herba* | MOL001955 | Heriguard | -6.96 |
| *Artemisiae Scopariae Herba* | MOL007260 | Isorhamnetin-3-mono-beta-D-glucoside | -6.85 |
| *Erigeron Breviscapus* | MOL012788 | Tiliroside | -9.68 |
| *Erigeron Breviscapus* | MOL004087 | 1,5-di-O-Caffeoylquinic acid | -8.14 |
| *Erigeron Breviscapus* | MOL002931 | Scutellarin | -7.95 |
| *Erigeron Breviscapus* | MOL006501 | Isochlorogenic acid b | -7.92 |
| *Erigeron Breviscapus* | MOL005922 | Acanthoside B | -7.68 |
| *Erigeron Breviscapus* | MOL001955 | Heriguard | -6.96 |
| *Lonicerae Japonicae Flos* | MOL003008 | Madreselvin B | -10.45 |
| *Lonicerae Japonicae Flos* | MOL003041 | Macranthoidin A | -10.15 |
| *Lonicerae Japonicae Flos* | MOL003042 | Macranthoidin B | -9.63 |
| *Lonicerae Japonicae Flos* | MOL003130 | Madreselvin A | -9.15 |
| *Lonicerae Japonicae Flos* | MOL000010 | Rhoifolin | -8.96 |
| *Lonicerae Japonicae Flos* | MOL003110 | Centauroside | -8.78 |
| *Lonicerae Japonicae Flos* | MOL003034 | Disacoside B | -8.72 |
| *Lonicerae Japonicae Flos* | MOL003038 | Fulvotomentoside A | -8.7 |
| *Lonicerae Japonicae Flos* | MOL000415 | Rutin | -8.43 |
| *Lonicerae Japonicae Flos* | MOL003051 | Scolymoside | -8.42 |
| *Lonicerae Japonicae Flos* | MOL001875 | Isochlorogenic,acid | -8.15 |
| *Lonicerae Japonicae Flos* | MOL003068 | 4,5-Dicaffeoylquinic acid | -8.03 |
| *Lonicerae Japonicae Flos* | MOL003106 | Akebiasaponin D | -7.96 |
| *Lonicerae Japonicae Flos* | MOL003010 | Quercetin-3-o-beta-D-glu | -7.89 |
| *Lonicerae Japonicae Flos* | MOL003118 | Isochlorogenic acid C | -7.87 |
| *Lonicerae Japonicae Flos* | MOL003054 | Eriodictyol-7-o-glucoside | -7.63 |
| *Lonicerae Japonicae Flos* | MOL004368 | Hyperin | -7.51 |
| *Lonicerae Japonicae Flos* | MOL000561 | Astragalin | -7.26 |
| *Lonicerae Japonicae Flos* | MOL000009 | Luteolin-7-o-glucoside | -7.22 |
| *Lonicerae Japonicae Flos* | MOL003066 | Neochlorogenic acid | -7.14 |
| *Lonicerae Japonicae Flos* | MOL003071 | Secologanoside | -7.11 |
| *Lonicerae Japonicae Flos* | MOL003108 | Caeruloside C | -7.11 |
| *Lonicerae Japonicae Flos* | MOL003067 | 3,4-Dicaffeoylquinic acid | -7.09 |
| *Lonicerae Japonicae Flos* | MOL003011 | Secologanate | -7.04 |
| *Lonicerae Japonicae Flos* | MOL001955 | Heriguard | -6.96 |
| *Lonicerae Japonicae Flos* | MOL003128 | Dinethylsecologanoside | -6.92 |
| *Lonicerae Japonicae Flos* | MOL003048 | Methyl chlorogenate | -6.9 |
| *Lonicerae Japonicae Flos* | MOL003073 | 8-epiloganin | -6.87 |
| *Lonicerae Japonicae Flos* | MOL003009 | Ochnaflavone | -6.85 |
| *Lonicerae Japonicae Flos* | MOL000007 | Cosmetin | -6.83 |
| *Lonicerae Japonicae Flos* | MOL000655 | Loganic acid | -6.83 |
| *Forsythiae Fructus* | MOL003332 | Forsythoside C | -10.46 |
| *Forsythiae Fructus* | MOL003337 | Forsythoside F | -10.36 |
| *Forsythiae Fructus* | MOL003363 | Forsythoside B | -10.09 |
| *Forsythiae Fructus* | MOL003316 | β-hydroxyacteoside | -9.87 |
| *Forsythiae Fructus* | MOL003314 | Suspensaside B | -9.7 |
| *Forsythiae Fructus* | MOL003338 | Forsythoside G | -9.31 |
| *Forsythiae Fructus* | MOL003352 | Procyanidin | -9.07 |
| *Forsythiae Fructus* | MOL003333 | Acteoside | -8.99 |
| *Forsythiae Fructus* | MOL003284 | Caleolarioside A | -8.83 |
| *Forsythiae Fructus* | MOL003331 | Forsythiaside | -8.62 |
| *Forsythiae Fructus* | MOL003313 | Suspensaside A | -8.6 |
| *Forsythiae Fructus* | MOL002505 | C10230 | -8.45 |
| *Forsythiae Fructus* | MOL000415 | Rutin | -8.43 |
| *Forsythiae Fructus* | MOL003334 | Forsythoside D | -8.4 |
| *Forsythiae Fructus* | MOL003309 | Plantainoside A | -8.37 |
| *Forsythiae Fructus* | MOL000522 | Arctiin | -8.34 |
| *Forsythiae Fructus* | MOL003336 | Forsythoside E | -7.78 |
| *Forsythiae Fructus* | MOL000536 | Matairesinoside | -7.76 |
| *Forsythiae Fructus* | MOL003327 | Rengyoside C | -7.52 |
| *Forsythiae Fructus* | MOL004368 | Hyperin | -7.51 |
| *Forsythiae Fructus* | MOL000437 | Hirsutrin | -7.51 |
| *Forsythiae Fructus* | MOL000702 | Guajavarin | -7.49 |
| *Forsythiae Fructus* | MOL003305 | PHILLYRIN | -7.47 |
| *Forsythiae Fructus* | MOL002037 | Amentoflavone | -7.42 |
| *Forsythiae Fructus* | MOL000561 | Astragalin | -7.26 |
| *Forsythiae Fructus* | MOL003288 | Cornoside | -7.24 |
| *Forsythiae Fructus* | MOL003320 | (-)-Olivir | -7.22 |
| *Forsythiae Fructus* | MOL003355 | Olivil | -7.22 |
| *Forsythiae Fructus* | MOL003299 | Forsythide | -7.18 |
| *Forsythiae Fructus* | MOL003324 | Rengyoside A | -7.17 |
| *Forsythiae Fructus* | MOL003339 | Adoxosidic acid | -7.15 |
| *Forsythiae Fructus* | MOL003301 | Forsythidmethylester | -7.08 |
| *Forsythiae Fructus* | MOL013068 | Oroxindin | -7.07 |
| *Forsythiae Fructus* | MOL002503 | Cynanuriculoside A | -7.05 |
| *Forsythiae Fructus* | MOL003298 | Forsythialan B | -6.97 |
| *Forsythiae Fructus* | MOL000416 | Lariciresinol | -6.97 |
| *Forsythiae Fructus* | MOL001938 | Secoisolariciresinol | -6.85 |
| *Gardeniae Fructus* | MOL009038 | GBGB | -8.46 |
| *Gardeniae Fructus* | MOL000415 | Rutin | -8.43 |
| *Gardeniae Fructus* | MOL001878 | 4,5-Di-O-caffeoylquinic acid | -7.92 |
| *Gardeniae Fructus* | MOL006502 | 3,5-Di-O-caffeoylquinic acid | -7.71 |
| *Gardeniae Fructus* | MOL007244 | 3,4-di-o-caffeoylquinic acid | -7.66 |
| *Gardeniae Fructus* | MOL009547 | Desacetyl asperulosidic acid | -7.62 |
| *Gardeniae Fructus* | MOL000437 | Hirsutrin | -7.51 |
| *Gardeniae Fructus* | MOL001668 | Geniposidic acid | -7.02 |
| *Gardeniae Fructus* | MOL004557 | Geniposide | -6.97 |
| *Gardeniae Fructus* | MOL001955 | Heriguard | -6.96 |
| *Gardeniae Fructus* | MOL001661 | Scandoside methyl ester | -6.94 |
| *Gardeniae Fructus* | MOL001666 | Deacetyl asperulosidic acid methyl ester | -6.94 |
| *Gardeniae Fructus* | MOL007994 | Ilexoside A_qt | -6.94 |
