# Supplementary material for: Mitochondrial toxicity evaluation of traditional Chinese medicine injections with a dual in vitro approach
Source: Front Pharmacol. 2022 Nov 2;13:1039235. doi: 10.3389/fphar.2022.1039235 (PMC9667049; doi:10.3389/fphar.2022.1039235)
Supplement: Supplementary file 4 [file Table3.DOCX]

**Supplementary Table S3.** The effect of control compounds exposure on ATP depletion in HepG2 cells.

| **Compound** | **IC_50_-ATP** | **IC_50_-ATP** | **IC_50_-ATP** | **IC_50_-ATP** | **Mitochondrial Toxicity** |
| --- | --- | --- | --- | --- | --- |
|  | **Mean ± SD** | **Mean ± SD** | **Glu/Gal** | **Dose** | **Glu/Gal≥2** |
|  | **Glucose** | **Galactose** | **Ratio** | **Unit** | **Yes/No** |
| Rotenone | 0.3001 | 0.0021 | 142.9048 | μM | Yes |
| Oligomycin A | 0.0110 | 0.0001 | 110.0000 | μM | Yes |
| Antimycin A | 0.7204 | 0.0489 | 14.7321 | μM | Yes |
| Metformin | 94.9080 | 5.0570 | 18.7676 | mM | Yes |
| Tamoxifen | 6.3810 | 7.0550 | 0.9045 | μM | No |
| Digoxin | 0.2347 | 0.2609 | 0.9667 | μM | No |

**Note:** IC_50_ ATP-Glu/Gal≥2 indicates potential mitochondrial toxicity.
