# Supplementary material for: Mitochondrial toxicity evaluation of traditional Chinese medicine injections with a dual in vitro approach
Source: Front Pharmacol. 2022 Nov 2;13:1039235. doi: 10.3389/fphar.2022.1039235 (PMC9667049; doi:10.3389/fphar.2022.1039235)
Supplement: Supplementary file 5 [file Table4.DOCX]

**Supplementary Table S4.** The cytotoxicity of control compounds in HCT116 (WT, *SCO2* KO) cells.

| **Compound** | **IC_50_-Survival** | **IC_50_-Survival** | **IC_50_-Survival** | **IC_50_-Survival** | **Mitochondrial Toxicity** |
| --- | --- | --- | --- | --- | --- |
|  | **Mean ± SD** | **Mean ± SD** | **(*SCO2* KO)/WT** | **Dose** | **(*SCO2* KO)/WT≥2** |
|  | **WT** | ***SCO2* KO** | **Ratio** | **Unit** | **Yes/No** |
| Rotenone | 0.0187 | 34.6400 | 1850.4274 | μM | Yes |
| Metformin | 0.4151 | 224.3820 | 540.5493 | mM | Yes |
| Digoxin | 0.0937 | 0.1639 | 1.7496 | μM | No |

**Note:** IC_50_-Survival- (*SCO2* KO)/WT≥2 indicates potential mitochondrial toxicity.
